# In Vitro Studies of Lipopolysaccharide-Mediated DNA Release of Podovirus HK620

**DOI:** 10.3390/v10060289

**Published:** 2018-05-29

**Authors:** Nina K. Broeker, Franziska Kiele, Sherwood R. Casjens, Eddie B. Gilcrease, Anja Thalhammer, Joachim Koetz, Stefanie Barbirz

**Affiliations:** 1 Physikalische Biochemie, Universität Potsdam, Karl-Liebknecht-Str. 24-25, 14476 Golm, Germany; nina.broeker@uni-potsdam.de (N.K.B.); frkiele@uni-potsdam.de (F.K.); thalhamm@uni-potsdam.de (A.T.); 2 Division of Microbiology and Immunology, Department of Pathology, University of Utah School of Medicine, Salt Lake City, UT 84112, USA; sherwood.casjens@path.utah.edu (S.R.C.); eddie.gilcrease@path.utah.edu (E.B.G.); 3 Kolloidchemie, Universität Potsdam, Karl-Liebknecht-Str.24-25, Haus 25, 14476 Golm, Germany; koetz@uni-potsdam.de

**Keywords:** O-antigen specific phage, podovirus, HK620, lipopolysaccharide, in vitro particle opening, tailspike protein

## Abstract

Gram-negative bacteria protect themselves with an outermost layer containing lipopolysaccharide (LPS). O-antigen-specific bacteriophages use tailspike proteins (TSP) to recognize and cleave the O-polysaccharide part of LPS. However, O-antigen composition and structure can be highly variable depending on the environmental conditions. It is important to understand how these changes may influence the early steps of the bacteriophage infection cycle because they can be linked to changes in host range or the occurrence of phage resistance. In this work, we have analyzed how LPS preparations in vitro trigger particle opening and DNA ejection from the *E. coli* podovirus HK620. Fluorescence-based monitoring of DNA release showed that HK620 phage particles in vitro ejected their genome at velocities comparable to those found for other podoviruses. Moreover, we found that HK620 irreversibly adsorbed to the LPS receptor via its TSP at restrictive low temperatures, without opening the particle but could eject its DNA at permissive temperatures. DNA ejection was solely stimulated by LPS, however, the composition of the O-antigen dictated whether the LPS receptor could start the DNA release from *E. coli* phage HK620 in vitro. This finding can be significant when optimizing bacteriophage mixtures for therapy, where in natural environments O-antigen structures may rapidly change.

## 1. Introduction

Bacteriophages have come back into research focus in recent years because new antimicrobial tools are needed due to the global rise of antibiotic resistance [1]. However, the importance of these applications is not proportionate to the current poor understanding of the molecular mechanisms by which bacteriophages deliver their DNA into susceptible cells. In vitro mixing of purified phage particles with purified cell surface receptor molecules is very often sufficient to trigger genome ejection [2,3]. Kinetics of DNA release have been examined with a variety of methods, both in bulk and on the single particle level, with different model phages like siphoviruses λ, T5 or SPP1 and/or podoviruses P22 and T7 [4,5,6,7,8,9]. Compared to the time scales observed for in vivo genome transfer into the cell, in vitro experiments that only monitored DNA egress showed that the individual process of particle opening occurred much more rapidly. This illustrates–depending on the experimental set up, that these in vitro studies only monitor a subset of all events that orchestrate the DNA transfer into the cell. The majority of these studies have employed bacteriophages that recognize protein receptors.

However, many phages use non-protein receptors. Especially for those that come across diderm, i.e., gram-negative, cell wall architectures only limited data is available on interactions with outer membrane components other than proteins. Here, pathogenic *E. coli* are an important bacteriophage therapy target and reductionist laboratory model systems are required to develop efficient strategies for employing bacteriophages as part of the more complex gut microbiota [10,11]. Gram-negative-specific bacteriophages encounter the lipopolysaccharide (LPS) as the first extracellular layer and can also use it as a receptor [3,7,12,13,14]. The LPS molecule consists of two parts, the O-antigen polysaccharide, composed of variable repeating oligosaccharide units linked to an oligosaccharide core structure, and the more rigid lipid A that forms the distal part of the gram-negative outer membrane heterobilayer [15,16]. It was stated already in the 1970s that purified LPS preparations are capable of inactivating certain bacteriophages, i.e., reducing the number of infective particles in a preparation [17]. However, with the technical state of the art at the time, these experiments could not quantify the DNA egress from particles stimulated by LPS in vitro or assign it to defined molecular rearrangements in the tail parts.

How LPS initiates particle opening in vitro has been investigated for *Salmonella* phage P22. P22 is an O-antigen-specific podovirus with an icosahedral head filled with double stranded DNA and a short, non-contractile tail [18,19]. P22 has six trimeric tailspike proteins (TSP) that bind and hydrolyze LPS of the host bacteria [13]. In the first step of infection the TSP specifically recognize and enzymatically cleave the O-antigen [20]. This step is essential and allows the virion to adsorb to the LPS receptor [7]. As a consequence, the tail needle gp26 comes in close proximity to the more rigid lipid A part of LPS and it was hypothesized that this contact triggers opening of the particle [21]. In vitro DNA ejection from phage P22 was accomplished in about 90 min, similar to podovirus T7, but slower than the ejection from siphovirus 9NA using the same TSP-receptor system [7,8].

In this work we have analyzed in vitro particle opening in *E. coli* podovirus HK620. HK620 is a temperate lambdoid podovirus that infects *E. coli* with a serogroup O18A1 O-antigen [22,23,24]. The HK620 prophage is maintained in *E. coli* under the control of the transcription termination factor Rho [25]. HK620 has an icosahedral, symmetrical head of 59 nm width, its 38.3 kbp dsDNA genome is packed through a headful mechanism [26]. Virion structural proteins of HK620 resemble those of P22 in number and size [26], with a similar gp26 plug protein [27] and a structurally similar TSP [24] that specifically recognizes the O-antigen of its *E. coli* host [28]. We found that in vitro DNA release from phage HK620 was triggered by lipopolysaccharide (LPS) with a velocity similar to that found in other podoviruses. HK620 irreversibly adsorbs to its LPS receptor at restrictive low temperatures without opening the particle but can eject its DNA at permissive temperatures. However, we found that the composition of the O-antigen dictates whether the LPS receptor can start the DNA release, emphasizing that the LPS structure has a predominant influence on early steps of infection in O-antigen-specific phages. This is especially significant in bacteriophage therapy of gram-negative pathogens, where LPS-dependent host range alteration or phage resistances are important issues to understand.

## 2. Materials and Methods

### 2.1. Materials

Phage HK620 was kindly provided by Mireille Ansaldi from University of Marseille and was propagated on *E. coli* H TD2158 as described elsewhere [21] and has O-serotype O18A1 [23]. *E. coli* strains were purchased from the German collection of microorganisms and cell cultures (DSMZ): DSM 10809 (O18A:K1:H-), DSM 10797 (O18A1: K5:H7), DSM 10837 (O18B:K?:H14), DSM 10922 (O18B1:K?:H14). *E. coli* H TD2158 [22] (O18A1) was a kind gift from Alvin J. Clark and *E. coli* IHE3042 (O18A1:K5:H7) was kindly provided by Anja Siitonen from the National Institute for Health and Welfare, Finland. If not indicated otherwise, 50 mM Tris/HCl, pH 7.6, 4 mM MgCl_2_ was used as a standard buffer. The truncated HK620TSPΔN without an N-terminal particle-binding domain was used for all experiments, as the latter does not participate in O-antigen binding [24].

### 2.2. Deletion of ompA or ompC Genes from E. coli H TD2158

The *ompA* and *ompC* genes of *E. coli* H TD2158 were replaced by tetracycline and kanamycin resistance gene cassettes, respectively, using homologous recombination as described before for *Shigella* [29]. The *ompA* replacement was constructed by amplifying the TetRA module from strain UB-1766 [30] with the following oligonucleotides that have 3′-tails that allow recombinational replacement of *ompA* in *E. coli*: 5′-CGGGG TTTTTCTACCAGACGAT AACTTAAGCCTGCGGCTGAGTTACAACGCACCAAACACCCCCCAAAACC and 5′-GTGCTC GGCATAAGCCGAAGATATCGGTAGAGTTAATATTGAGCAGATCCACACAACCACACCACACCAC, transforming TD2158 with the amplified DNA and selecting for tetracycline resistance. The *ompC* replacement was constructed by using primers 5′-GGCCCGACGGTAATAT and 5′-TTGCTGGAAATTATGC to amplify an approximately 2 kbp DNA fragment that includes the kanamycin cassette that replaces the *ompC* gene in *E. coli* K-12 strain JM2203 DNA [31], and using this fragment to replace the *ompC* gene in strain TD2158.

### 2.3. Preparation of LPS, Lipid A, PS and TSP-Digested LPS

*E. coli* cells of strain H TD2158 were grown overnight at 37 °C in LB medium. Specific staining showed that all other *E. coli* strains used in this study expressed capsules described earlier as K1 or K5. These were cultured at 18 °C in order to minimize capsule synthesis. LPS purification, as well as TSP-digested LPS, have been described [21]. All LPS preparations were highly purified and spectroscopically analyzed for being free of membrane protein contaminants that might interact with the phage particle. Lipid A and O-antigen polysaccharide were obtained from the purified LPS by the acid hydrolysis method [32].

### 2.4. DNA Ejection and Phage Inhibition Experiments

In vivo inhibition of phage HK620 by LPS preparations was determined in a plaque forming assay. 25 µg mL^−1^ LPS and about 5000 phage HK620 plaque forming units (pfu) per mL in 50 mM Tris pH 7.6, 4 mM MgCl_2_ were incubated in glass tubes at 37 °C or 45 °C for indicated times. Then, 100 µL of the solutions were plated on *E. coli* H TD2158.

In vitro DNA ejection was monitored via Yo-Pro^®^ fluorescence as described [21] by incubating 6.7 × 10^9^ pfu mL^−1^ HK620 phages with LPS from *E. coli* H TD2158, at different temperatures. At the end of each experiment, DNase I (10 µg/mL) was added as a control for DNA accessibility. Kinetic traces were fitted to Equation (1) describing two sequential first-order processes [33] (see Figure S1 for details on the fitting procedure):

(1) $$DNA\left(t\right)=A_{0}\cdot \left(1-\frac{1}{k_{1}-k_{2}}\cdot \left(k_{1}\cdot e^{\left(-k_{2}\cdot t\right)}-k_{2}\cdot e^{\left(-k_{1}\cdot t\right)}\right)\right)$$

### 2.5. LPS Morphology Studies

LPS was analyzed by dynamic light scattering (laboratory built apparatus consisting of a 0.5 W diode-pumped continuous-wave laser (λ 532 nm; Cobolt Samba, Cobolt AB, Stockhom, Sweden), a high quantum yield avalanche photo diode and an ALV 7002–25 ns correlator (ALV-GmbH, Bargteheide, Germany at 90 deg scattering angle)). Different LPS concentrations were investigated (0.01 mg mL^−1^, 0.1 mg mL^−1^, 0.2 mg mL^−1^, 1 mg mL^−1^) in a 3 mm quartz cell in standard buffer. To determine Stokes radii, 50 or 100 correlation functions were measured in 8.4 s intervals at 37 °C ± 0.1 °C. Average concentration independent Stokes radii were calculated with CONTIN [34] using dynamic viscosity and the refractive index of the buffer. TEM images were obtained on carbon-coated copper grids in a JEOL JEM 1011 (JEOL, Freising, Germany), operating at 80 kV accelerating voltage.

## 3. Results

### 3.1. Bacteriophage HK620 Releases Its DNA Upon LPS Incubation In Vitro

**Figure 1:**
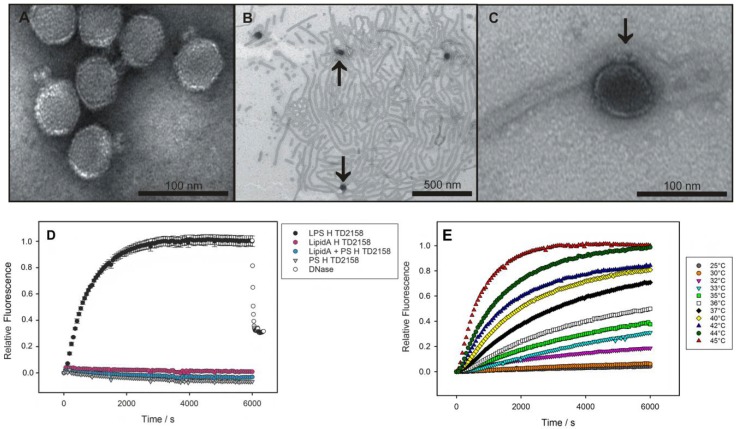
LPS-mediated DNA-release from phage HK620 observed in vitro. (**A**) Transmission electron microscopy images of phage HK620 particles (1 × 10^10^ pfu mL^−1^) stained with 1% (*w*/*v*) uranyl acetate and pre-incubated with 0.24 mg mL^−1^ LPS from *E. coli* H TD2158 (O18A1) at 37 °C (**B**,**C**). LPS from *E. coli* H TD2158 (O18A1) forms ribbon-like aggregates (**B**) and phages are attached to these LPS structures (arrows). (**D**) DNA ejection from phage HK620 (6.7 × 10^9^ pfu) triggered by 16.7 μg/mL *E. coli* H TD2158 (O18A1) LPS in the presence of the fluorescent DNA-binding dye Yo-Pro at 45 °C (black circles). Ejected DNA could be digested by adding DNase after 6000 s (white circles). Controls contained an *E. coli* H TD2158 (O18A1) lipid A preparation (pink circles), an O-antigen polysaccharide preparation lacking lipid A (grey triangles) or a mixture of both (blue circles). Standard deviations were obtained from three independent experiments and do not exceed 5% of the total fluorescence signal. (**E**) DNA ejection of HK620 at different temperatures (same conditions as in **D**).

Native HK620 particles were analyzed with transmission electron microscopy (TEM) (Figure 1A). After incubation with purified LPS from *E. coli* H TD2158 (O18A1), emptied particles attached to the LPS and appeared dark in the negative stain, indicating that the virions were empty and devoid of DNA (Figure 1B,C). In addition, in agarose gels notably increased amounts of free phage DNA were observed when HK620 was treated with its host cell LPS receptor (Figure S2). We therefore conclude that host cell LPS preparations can trigger in vitro DNA ejection from HK620 phage particles.

As a convenient in vitro experimental set-up, bacteriophage particle opening can be monitored via an increasing fluorescence signal produced by the ejected genome in presence of the DNA-specific dye Yo-Pro [7,8,21]. When we incubated bacteriophage HK620 with LPS from its *E. coli* H TD 2158 (O18A1) host we observed an increasing fluorescence signal (Figure 1D). This signal was saturated within about 3000 s and could be decreased by the addition of DNase, furthermore proving that the ejected DNA had provoked the signal increase. The process was highly specific, neither polysaccharide alone, lipid A lacking the O-antigen part, or a mixture of these two LPS components led to an increase in fluorescence. We therefore conclude that HK620 is strictly O-antigen dependent on the in vitro particle opening route. Both the amount of DNA ejected and the velocity of the process was temperature dependent. We found the fastest ejection kinetics at 45 °C (Figure 1E) whereas at temperatures above 45 °C the ejection efficiency clearly decreased (Figure S3). DNA concentration increase was fitted to two sequential first-order processes for experiments in a temperature range between 32 and 45 °C (Figure S1). The Arrhenius temperature dependencies of the rate constants resulted in activation barriers for the two steps of 125 kJ mol^−1^ and 156 kJ mol^−1^, respectively (Figure S4).

### 3.2. LPS Is the Receptor Sufficient for HK620 Host Cell Infection

**Figure 2:**
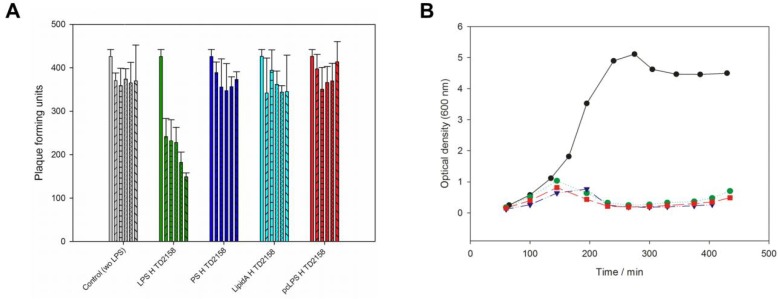
Phage HK620 in vivo infection assays. (**A**) In vivo plaque forming assay after incubation of 4.5 × 10^3^ pfu mL^−1^ HK620 particles with 25 µg mL^−1^ LPS from *E. coli* H TD2158 LPS (green). After 0, 10, 30, 120, 240, 300 min (individual bars in each experiment) the reaction mix (450 pfu) was plated on *E. coli* H TD2158 and plaques were determined after overnight incubation at 37 °C. Controls: Phage buffer (grey), 25 µg mL^−1^ polysaccharide (dark blue), lipid A (cyan) or *E. coli* H TD2158 LPS pretreated with HK620TSP (red). Standard deviations are from three independent experiments. (**B**) Time course of OD_600_ of cultures at 37 °C of *E. coli* strains H TD2158 (green), H TD2158 ΔOmpA (purple) or H TD2158ΔOmpC (red) after addition of phage HK620 at OD 0.3 with a multiplicity of infection of 0.1. No phage was added to a control with *E. coli* H TD2158 (black).

If LPS specifically provoked DNA release from phage HK620 in vitro, it should also be able to inactivate phage particles prior to in vivo infection. Incubation of phage HK620 with its host LPS prior to plating decreased the number of plaque forming units (pfu) on host bacteria to 35% of the initial value (Figure 2A). *t*-Test analysis showed that this decrease in infectivity is significant (α < 0.05). In the control, the number of pfu was stable within the experimental time frame. We also incubated phage HK620 with lipid A or an O18A1 O-antigen polysaccharide preparation lacking the lipid part; however, no significant decrease in infectivity could be detected. This is in agreement with the fact that these LPS components alone were also unable to trigger DNA ejection in vitro (cf. Figure 1). Thus, intact LPS molecules must be present to inhibit HK620 infectivity. To check whether any outer membrane protein (Omp) was essential for HK620 infection we deleted the OmpA or OmpC encoding genes in *E. coli* H TD2158 (O18A1) by homologous recombination. HK620 had the same infectivity on these two mutant strains as wild types and cleared liquid cultures of these strains within the same time frame (Figure 2B). Hence, the deletion of OmpA or OmpC encoding genes had no effect on infectivity and these proteins can be excluded as outer membrane receptors for phage HK620.

It is important to note here that all LPS preparations used in the inhibition tests or in the in vitro experiments were free from outer membrane proteins (Omps). Moreover, during HK620 preparation no binding of phage particles to any protein component from the *E. coli* outer membrane was observed. This is in contrast to *Shigella* phage Sf6 that copurified with Omps [35]. Taken together these results support the notion that host cell LPS is the only receptor necessary to initiate DNA release of HK620 in vitro.

### 3.3. O-Antigen Specificity of TSP Guides Phage HK620 Host Specificity

**Figure 3:**
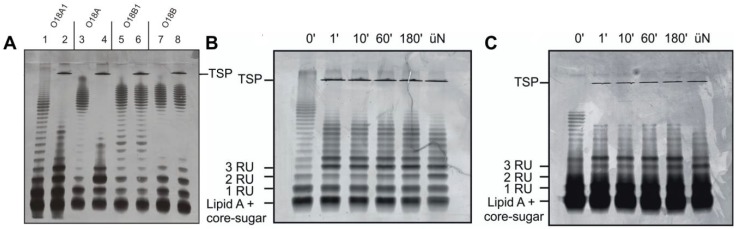
Activity of HK620 tailspike protein on *E. coli* LPS from different O-serogroups. (**A**) Purified LPS (O-serogroup given in parentheses) from *E. coli* H TD2158 (O18A1) (lanes 1, 2), DSM 10809 (O18A) (lanes 3, 4), DSM 10922 (O18B1) (lanes 5, 6) and DSM 10837 O18B (lanes 7, 8) were analyzed on 15% silver-stained SDS-PAGE before (lanes 1, 3, 5, 7) and after (lanes 2, 4, 6, 8) overnight incubation with HK620TSP. The HK620TSP protein band is indicated. (**B**,**C**) 0.5 mg mL^−1^ LPS from *E. coli* H TD2158 (**B**) or IHE3042 (**C**) were digested with 50 µg mL^−1^ HK620TSP at 37 °C for 1, 10, 60, 180 min and overnight. Positions of TSP and LPS with different repeat unit (RU) chain lengths are indicated. üN: over night.

Tailspike proteins (TSP) on LPS recognizing phages confer O-antigen specificity to the infection process [7,36,37]. TSP are modular proteins consisting of an N-terminal particle binding domain and a large C-terminal O-antigen interaction domain. Therefore, for HK620TSP an N-terminally truncated variant was used throughout all experiments. To test whether the tailspike interaction is an essential step in HK620 particle opening, we investigated several *E. coli* LPS preparations both for TSP interactions and their capacity to provoke DNA release. Purified HK620TSP recognizes and enzymatically cleaves the O-antigen of the immunological serogroup O18A1, which contains a hexasaccharide repeat structure [23,24]. We purified LPS from bacteria of various O18 serogroups (Figure S5) [38] and analyzed changes of the LPS ladder-like pattern in SDS-PAGE when incubated with HK620TSP. Whereas longer chain length fragments of O18A1 and O18A LPS disappeared and shorter ones accumulated (Figure 3A, lanes 1–4), no changes in the LPS migration pattern were observed for O18B and O18B1 LPS (Figure 3A, lanes 5–8). Thus, HK620TSP tolerated the lack of branching glucose in the O18A serogroup O-antigen as found on the *E. coli* strain DSM10809, while different glycosidic linkages that occur in the O18B serogroups prohibited binding. HK620TSP rapidly cleaved the O-antigen from different strains containing the O18A1 type O-polysaccharide (Figure 3B,C). Long chains already disappeared after one minute of incubation with HK620TSP at 37 °C; no notable changes in the distribution pattern of the accumulating short chains were observed over extended time frames. These O-antigen chain shortening velocities were found irrespective of the mean chain length in the initial preparation. Accordingly, the tailspike protein alone cleaved the LPS from the O18A1 O-antigen containing strain *E. coli* IHE3042 [28] in the same time; however, it contained a notably higher number of short chains and had a smaller mean O-antigen chain length when compared to the host strain *E. coli* H TD2158 (Figure 3C).

### 3.4. O-Antigen Chain Length Determines the Properties of the LPS Receptor

**Figure 4:**
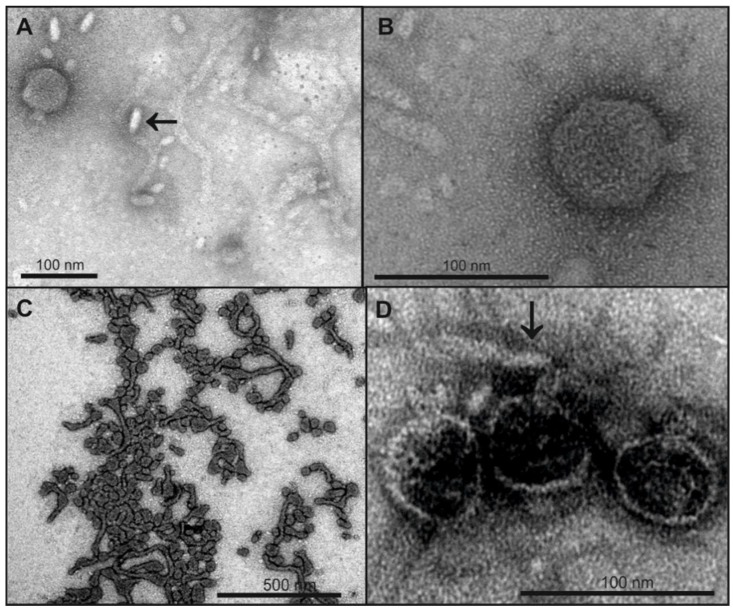
Legend: TEM micrographs of phage HK620 before and after incubation with non-host *E. coli* LPS. 1 × 10^10^ pfu mL^−1^ HK620 particles were stained with 1% (*w*/*v*) uranyl acetate after overnight incubation at 37 °C with 0.24 mg mL^−1^ LPS from *E. coli* IHE3042 (O18A1) (**A**,**B**) or *E. coli* DSM 10809 (O18A) (**C**,**D**). For IHE3042 (O18A1) with short O-antigen chains mainly small LPS structures are visible (arrow in **A**) and HK620 particles are not attached to these structures (**B**), whereas on DSM 10809 (O18A) LPS with longer O-antigen chains empty HK620 particles are attached (**D**, arrow).

LPS preparations with O18A or O18A1 O-serogroups from non-host *E. coli* strains were tested for their ability to inactivate HK620 phage particles. They only differ in a single α-d-Glc*p*-(1→6)-branch linked to the reducing end GlcNAc that is lacking in the O-polysaccharide repeat unit of O18A strains [38]. In contrast to the LPS isolated from the HK620 host strain TD2158 (O18A1) that contained long tubular structures (cf. Figure 1B), TEM micrographs showed that the LPS preparations from the *E. coli* DSM10809 (O18A) contained shorter filaments, for LPS from *E. coli* IHE3042 (O18A1) even smaller and only rod-like structures were found (Figure 4A,B). Accordingly, most HK620 particles were not attached to IHE3042 LPS (O18A1) and all observed particles remained intact (Figure 4A,B). However, upon mixing phage with DSM10809 LPS (O18A), empty particles occurred (Figure 4D). To determine whether aggregate morphology was an effect of the TEM sample preparation, we used dynamic light scattering (DLS) to estimate average hydrodynamic sizes of LPS aggregates in solution (Table 1).

We found that all calculated Stokes radii were independent of LPS concentration in the used range. LPS preparations of the HK620 host *E. coli* H TD2158 (O18A1) had Stokes radii of about 95 nm. For *E. coli* DSM10809 LPS (O18A), slightly reduced radii of about 70 nm were found, similar to *E. coli* IHE3042 LPS (O18A1) with a smaller Stokes radius of 65 nm. The *E. coli* IHE3042 LPS (O18A1) showed small structures on the EM grid and rarely formed rods (cf. Figure 4A arrow). This is in agreement with the appearance of this LPS on SDS gels, where it showed a high amount of short, i.e., rough and semi-rough, chains (cf. Figure 3). TSP-digested LPS also showed a clear reduction in Stokes radius (54 nm) compared to untreated *E. coli* H TD2158 LPS (O18A1) (95 nm) (Table 1).

**Figure 5:**
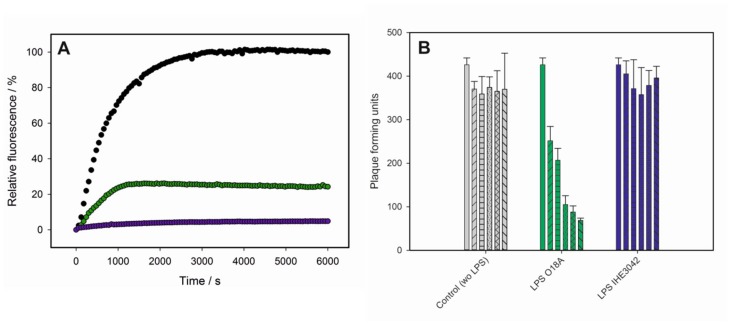
Interaction of phage HK620 with non-host *E. coli* strains. (**A**) DNA ejection from phage HK620 (6.7 × 10^9^ pfu) in the presence of the fluorescent DNA-binding dye Yo-Pro at 45 °C triggered by LPS (16.7 μg/mL) with O-antigen serotype O18A1 from *E. coli* H TD2158 (O18A1) (black) or *E. coli* IHE3042 (O18A1) (green) or serotype O18A from *E. coli* DSM10809 (O18A) (purple). (**B**) In vivo plaque forming assay after incubation of 4.5 × 10^3^ pfu mL^−1^ HK620 particles with 25 µg mL^−1^ LPS from *E. coli* DSM10809 (O18A) (green) or *E. coli* IHE3042 (O18A1) (blue). After 0, 10, 30, 120, 240, 300 min (individual bars in each experiment) the reaction mix (450 pfu) was plated on *E. coli* H TD2158 (O18A1) and plaques were determined after overnight incubation at 37 °C. Standard deviations are from three independent experiments.

In vitro DNA ejection of HK620 could be observed in fluorescence using *E. coli* DSM10809 LPS (O18A) as a receptor, although with a lower efficiency than found for the host LPS (Figure 5A). By contrast, LPS preparations of *E. coli* IHE3042 (O18A1) did not provoke DNA release. It is important to note here that HK620 was unable to successfully infect both *E. coli* DSM10809 (O18A) and *E. coli* IHE3042 (O18A1) strains tested and only formed plaques on its host strain *E. coli* H TD2158 (O18A1). However, when plaque formation on this strain was monitored after incubation with LPS from the two non-host strains, *E. coli* DSM10809 LPS (O18A) was found to significantly (α < 0.05) decrease HK620 pfu to 16% of the initial value (Figure 5B). In contrast, *E. coli* IHE3042 LPS (O18A) was unable to inactivate HK620 phage particles and could not provoke a decrease in the number of pfu on host cells (Figure 5B). In summary, we found that O-antigen serotype alone does not determine whether a given LPS preparation acts as a trigger for DNA release in vitro. Rather, our data suggests that a minimum LPS particle size is needed to inactivate HK620 and trigger DNA ejection.

### 3.5. Phage Adsorption in Competition with TSP-Mediated O-Antigen Cleavage

**Figure 6:**
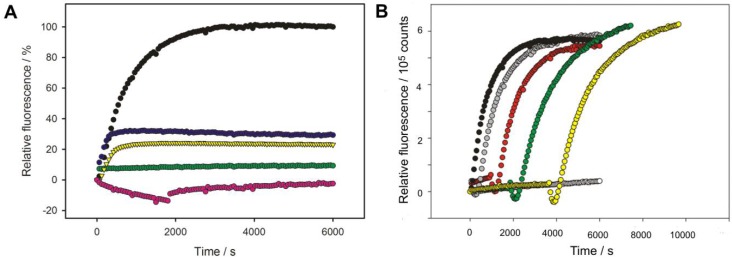
Phage adsorption and TSP-mediated O-antigen cleavage. DNA ejection from phage HK620 (6.7 × 10^9^ pfu) triggered by 16.7 μg/mL *E. coli* H TD2158 (O18A1) LPS in the presence of the fluorescent DNA-binding dye Yo-Pro at 45 °C (black circles). (**A**) Purified HK620TSP (3 nM) was added after 2 min (yellow triangles) or 5 min (blue circles). Controls: LPS digested with 0.3 nM HK620TSP for 30 min in the cuvette prior to adding phage (pink; A negative signal is due to correction for HK620 phage particle Yo-Pro staining over the full time course of the experiment) or LPS digested overnight with HK620TSP (green). (**B**) The mixture was kept in the cuvette at the restrictive temperature of 25 °C, where no ejection occurred (white) and only heated up to 45 °C after 2 (grey), 15 (red), 30 (green) or 60 min (yellow).

HK620 TSP digestion of the O-antigen receptor occurred rapidly within minutes at 37 °C when analyzed on SDS gels (cf. Figure 3). We monitored HK620 particle opening in the presence of *E. coli* H TD2158 (O18A1) LPS via DNA fluorescence as described above (cf. Figure 1) and deprived the system of its O-antigen receptor by adding HK620TSP after two minutes (Figure 6A). This remarkably decreased the amount of ejected DNA to about 20%. If HK620TSP was only added after five minutes, about 30% of the initial signal was obtained. No DNA ejection at all was found when LPS pre-incubated overnight or for 30 min with HK620 TSP was added to HK620 phage particles. This means that HK620 adsorption to LPS requires an intact O-antigen receptor at an early time point.

To further test the irreversible adsorption of HK620 particles we mixed them with LPS at a restrictive temperature of 25 °C where no DNA ejection occurred (cf. Figure 1). We monitored Yo-Pro fluorescence as above in the mixtures for different times and then heated the fluorescence cuvette to the permissive temperature of 45 °C (Figure 6B). After an initial loss of fluorescence signal due to the temperature increase, we found that the full signal was recovered irrespective of the waiting time at 25 °C and even after 60 min all phage particles in the mixture were still able to eject their DNA. Given that cleavage of the O-antigen receptor by the TSP is fast we therefore conclude that the initial LPS adsorption of HK620 can occur rapidly and irreversibly.

## 4. Discussion

For phage HK620, LPS is necessary and sufficient to trigger in vitro DNA ejection and therefore is the sole receptor of this O-antigen-specific podovirus. This has been shown in this work by (i) LPS triggered DNA release in an in vitro fluorescence assay, (ii) in electron microscopy where LPS incubation showed particles that had released their DNA and (iii) in LPS-mediated inhibition of infective particles. Like bacteriophage P22, HK620 needed no additional protein receptor for in vitro DNA release [21], this is in agreement with the fact that deletion of OmpA or OmpC in its *E. coli* host did not influence HK620 infectivity. Even more, all LPS preparations used in the experiments described were extensively treated with protease to remove all protein content. TSP molecules on the phage particles are responsible for the specificity of the recognition process and no in vitro DNA release was found with LPS from the O18B serogroups. However, TSP O-serogroup specificity did not dictate alone whether LPS was able to trigger DNA ejection from HK620 phage particles in vitro. For example, intact phage particles could be detected on agarose gels after LPS incubation, and LPS treatment reduced but did not completely abolish phage infectivity in the host bacterial plating assay.

### 4.1. Role of O-Antigen Receptor Composition

For podovirus HK620 we observed that the LPS preparation must contain enough molecules equipped with long O-antigen chains to trigger DNA egress in vitro. If the LPS receptor did not contain enough long chains or was depleted from the O-antigen by enzymatic cleavage with HK620TSP, no ejection could occur. The proportion of molecules in the preparation with long O-antigen chains is also typically reflected in different LPS aggregate morphologies in electron microscopy [15]. LPS isolated from natural sources leads to molecular mixtures with complex aggregation behavior, the principles of which are not well understood. Here, hydrophobic thickness of homogeneous LPS bilayers largely depends on the composition of the lipid A molecules as well as the number, order and chain length of acyl chains [39]. Accordingly, we observed varying aggregate sizes with LPS from different bacterial strains of the O18A group (i.e., O18A and O18A1, see Figure S5), although all O-antigens were specifically recognized by HK620TSP. Here, we observed three situations: (i) We found smooth LPS in the host strain *E. coli* H TD2158, with long O-antigen chains forming ribbons and filaments able to initiate phage HK620 ejection. Similar morphologies were also observed for *S.* Typhimurium LPS preparations that triggered DNA release from phage P22 [7]. (ii) A mixture of long chains and short chains was present in *E. coli* DSM10809 that could in part provoke DNA release from phage HK620, or (iii) a high concentration of rather short O-antigen chains dominated LPS preparations from *E. coli* IHE3042, that were unable to act as a receptor for HK620.

These results illustrate that even if the correct O-antigen receptor is present and can be addressed by the TSP, this will not ultimately lead to DNA ejection from an O-antigen-specific phage. Rather, this emphasizes that infection is not only O-antigen specific but is also tightly linked to the LPS composition that is dynamic with bacterial growth conditions and metabolic state [40,41,42]. Here, it is important to emphasize that many non-lethal mutations affecting O-antigen synthesis apparatus of gram-negative bacteria can interfere with bacteriophage infection [43]. Moreover, it has been found that O-antigen chain length can be under epigenetic control in *S. typhimurium*, which transiently blocked infection by phages P22, 9NA and Det7 [44]. In the case of HK620, these dynamic changes in the cell envelope of *E. coli* are most probably the reason why the phage was not able to infect alternative O18A strains. Both strains, *E. coli* IHE3042 and *E. coli* DSM10809, were protected with capsules at the permissive 37 °C replication temperatures of HK620. However, both strains produced sufficient amounts of smooth LPS at 18 °C (see Materials and Methods), a temperature where HK620 particles could not open.

### 4.2. In Vitro Particle Opening

In vitro experiments are useful in testing how well an LPS preparation can mimic the membrane environment that the phage encounters in vivo. Although they cannot show how phage proteins and genetic material enter the cytosol of a living bacterial cell, they provide a reductionist approach to the mechanisms in the bacteriophage infection machine. In vivo, to transport its DNA over all parts of the gram-negative cell envelope, P22 particles extend their tails to form tubular structures, as found in other gram-negative-specific phages [45,46,47]. These extensions at the tip of the tail are most probably formed by P22 ejection proteins that must leave the particle at an early time point after opening. Accordingly, initial LPS contacts leading to rearrangements in the tail and to particle opening are crucial to start genome delivery.

We monitored kinetics of in vitro HK620 particle opening with a DNA-sensitive fluorescence assay. The DNA egress can be described by two sequential first-order equations, suggesting that our bulk manual mixing experimental setup resolved two steps of the process. Remarkably, similar kinetics of in vitro DNA ejection were found for other O-antigen-specific podoviruses like P22 and Sf6 (Figure S6). These kinetics are dominated by conformational rearrangements in the phage particle, as both the receptor association and the DNA ejection steps are fast [7,48,49]. We propose that the LPS enzymatic cleavage by TSP and the subsequent positioning of the phage close to the rigid lipid A part of the LPS receptor might constitute a first fast step. In a second step, the membrane must confer an additional trigger to induce conformational change in the tail to open the particle. For HK620 we could show that LPS of a certain length is necessary for the first step of positioning the phage (see above). After this step a minimum thermal energy is crucial to trigger the ejection process and we could show that bacteriophage HK620 “waits” until this energy is provided by rising temperatures. Although we have only analyzed O-antigen cleavage velocities at 37 °C, we can assume that TSP cleaves long O-antigen chains very rapidly also at lower temperatures [50,51]. We thus hypothesize that the phage is already irreversibly bound to the LPS at 25 °C and that the TSP-mediated O-antigen digest has already been completed.

A very important finding for O-antigen-specific bacteriophages is that in order to act as a functional receptor the O-antigen must be part of a membrane and that the isolated O-polysaccharide will not trigger particle opening in vitro [7,21]. Here, enzymatic cleavage is necessary to enable binding to LPS, which illustrates the double-acting role of the LPS receptor. We hypothesize that O-antigen cleavage helps in positioning the phage onto the membrane where a second signal starts conformational rearrangements in the tail. Otherwise, the phages would also be able to infect rough strains if binding to a core or lipid A component was sufficient, like for example in phage T7 [8]. In the latter, a multivalent contact to core LPS turns down the short tail fibers towards the cell surface, which opens the gp12 nozzle [52]. The only tail protein in bacteriophage HK620 that can closely approach the membrane surface for sensing a mechanical signal starting tail rearrangement, is the tail needle gp26 that was proposed to serve as a membrane sensor for particle opening in bacteriophage P22 [21]. Gp26 forms an α-helical coiled-coil and binds to the tail machine with its N-terminus [53]. The C-terminal end can then make contact with the cell surface and different structures can been found for the P22-like podoviruses: Coiled-coil like in P22 and HK620 [27] and knob-like in P22-like phages Sf6 [54] and HS1 [30]. When the phage gets close enough to the membrane we hypothesize that its gp26 plug pushes against the rigid LPS parts, which opens the particle for DNA release. The dimensions of the host LPS receptor parts and the HK620 phage tail would well fit to such a model (Figure S7). Accordingly, a mechanical LPS contact rather than a specific binding event would be the second step in the phage particle opening process [21]. This is in agreement with the fact that the LPS-contacting C-terminal domain of gp26 can be exchanged between phages and is not host species specific [30]. It is well conceivable that this step is controlled not only by successful adsorption to the outer membrane but also by a thermal energy barrier in order to prevent premature DNA loss from the phage particles [55]. This might be reflected in the rather similar activation energy barriers of the phage particle found for opening processes in vitro (Table 2), suggesting conserved conformational rearrangement steps in the phage tail, irrespective of the receptor type [6]. It has been proposed that portal opening is rate limiting and dominating the observed in vitro processes, it is well conceivable that for all podoviruses studied so far and also for HK620 in this study, very similar DNA egress velocities have been measured [8,21,55].

### 4.3. Properties of the LPS Receptor Influencing In Vitro Particle Opening

Also, upon in vivo infection the phage might need to encounter specifical dynamic regions of the bacterial membrane. Especially Omps might play a key role as experimental and theoretical studies showed that they may influence the LPS membrane thickness and structure [57,58]. The O-antigen-specific *Shigella* podophage Sf6 could only be inactivated in vitro by a mixture containing both LPS and OmpA or C [35]. Sf6 was found to copurify with outer membrane vesicles containing Omps, and in contrast to HK620, less efficiently infected a mutant *S. flexneri* host lacking Omp A or Omp C [35]. Apparently the specific composition of the LPS of its *Shigella* host requires a different particle interaction strategy. This is illustrated on the one hand by the special structure of the Sf6 gp26 needle with a C-terminal knob that is lacking in P22 and HK620 [54]. Future studies should analyze which factors, for example Omps, influence LPS membrane dynamics. Several phages show preferential infection at bacterial poles and/or need protein receptors on the membrane [59]. It remains to be elucidated for each case whether these membrane proteins solely establish a specific binding contact or whether they could also provide the surrounding membrane with specific biophysical properties, i.e., a certain membrane thickness or fluidity that could be sensed by parts of the phage tail apparatus.

In principle, the thermal barriers that we observed for the HK620 DNA ejection process in vitro could have two origins: First, a temperature-dependent destabilization of the tail complex leading to an ejection-competent particle at a permissive temperature. Recently, it was shown for the podovirus T7 that tapping the capsid wall with an oscillating atomic force microscope cantilever could trigger rapid DNA ejection via the portal, illustrating that a conformational switch can be mechanically activated by external forces [49]. Second, temperature-dependent structural transitions might also be present in the LPS aggregates that act as an in vitro receptor. Here, rising the temperature may influence the biophysical properties of the LPS glycolipid preparation, for example, enhanced dynamics and disorder of LPS aggregate packing. In the case of HK620, in certain temperature ranges we observed that small temperature changes led to a large increase of ejected DNA (cf. Figure 1). Hypothesizing that the LPS receptor undergoes phase transitions with temperature, the yield of successfully ejected particles would thus not linearly increase with temperature. Furthermore, molecular modeling analyses of *E. coli* LPS showed that smooth LPS was less densely packed and more dynamic than rough LPS [60]. O-antigen-specific phages thus might locally alter the biophysical properties of their receptor upon shortening their O-antigen with their TSP.

## Figures and Tables

**Table 1:** Stokes radii of lipopolysaccharide solutions estimated from dynamic light scattering.

Origin of LPS Preparation ^a,b^	Stokes Radius/nm
*E. coli* H TD2158 (O18A1) *	96
TSP digested, *E coli* H TD2158 (O18A1)	54
*E. coli* IHE3042 (O18A1)	66
*E. coli* DSM10809 (O18A) *	68

^a^ The O-serotype is given in parentheses. ^b^ An asterisk marks the ability of this LPS to elicit DNA release from phage HK620 in vitro.

**Table 2:** Activation barriers found for in vitro bacteriophage particle opening.

Bacteriophage	Activation Energy/kJ mol^−1^	Reference
HK620 ^1^	156	this work
P22	171	[7]
9NA	222	[7]
λ	109	[6]
T5	176	[56]
SPP1	125	[6]

^1^ HK620: E_A1_ = 156 kJ mol^−1^, E_A2_ = 125 kJ mol^−1^.

## Supplementary Materials

Supplementary materials can be found at http://www.mdpi.com/1999-4915/10/6/289/s1.
