# Supplementary material for: In Vitro Studies of Lipopolysaccharide-Mediated DNA Release of Podovirus HK620
Source: Viruses. 2018 May 29;10(6):289. doi: 10.3390/v10060289 (PMC6024685; doi:10.3390/v10060289)
Supplement: Supplementary file 1 [file viruses-10-00289-s001.pdf]

Figure S1: Kinetic analysis of DNA ejection from bacteriophage HK620... 2

Figure S2: DNA ejection from HK620 phage analyzed on agarose gels 3

Figure S3: LPS-mediated DNA-release from phage HK620 observed *in vitro* at temperatures above 45 °C. 4

Figure S4: Temperature dependencies of HK620 DNA ejection rate constants 5

Figure S5: O18 serogroups 6

Figure S6: Comparison of DNA ejection velocities of O-antigen specific podoviruses HK620, P22 and Sf6 7

Figure S7: Dimensions of glycolipid receptors and tail protein components 8

Figure S1: Kinetic analysis of DNA ejection from bacteriophage HK620

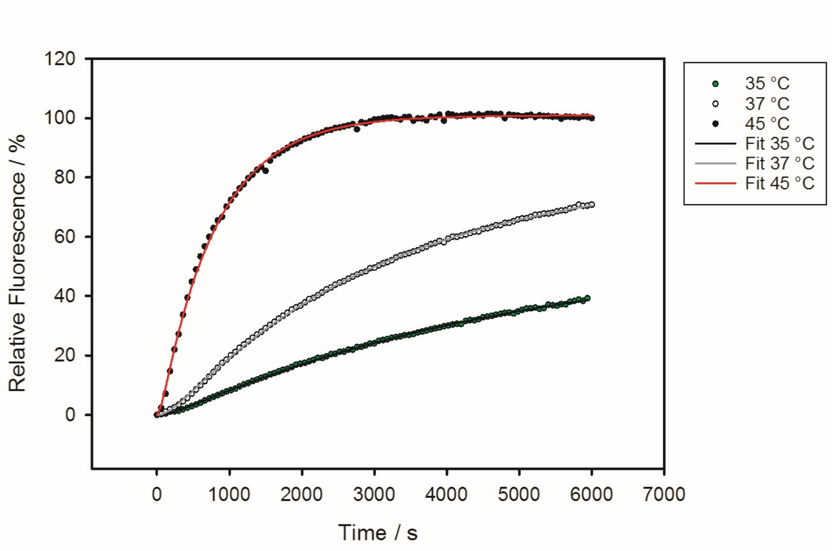

All kinetic traces (*cf.* Figure 1E) of Yo-Pro fluorescence increase measured in mixtures of bacteriophage HK620 and LPS were fitted to equation (1) describing two sequential first-order processes:

$$DNA(t) = A_0 \cdot \left(1 - \frac{1}{k_1 - k_2} \cdot \left(k_1 \cdot e^{(-k_2 \cdot t)} - k_2 \cdot e^{(-k_1 \cdot t)}\right)\right), \quad (1)$$

All fits were obtained with the program Solver implemented in Excel using the sum of squared residuals (SSR) as optimality criterion [33]. Exemplary fit lines are given in the above figure for three temperatures. Results for all temperatures are tabulated below:

Temperature / °C	k_1 / 10^{-3} s^{-1}	k_2 / 10^{-3} s^{-1}	SSR / $\%^2$
33	0.16	1.77	14.64
35	0.20	4.82	5.38
36	0.28	3.34	6.60
37	0.32	5.20	8.80
40	0.48	6.93	25.29
42	0.63	13.12	49.93
44	0.68	19.53	59.48
45	1.30	20.82	60.73

Figure S2: DNA ejection from HK620 phage analyzed on agarose gels

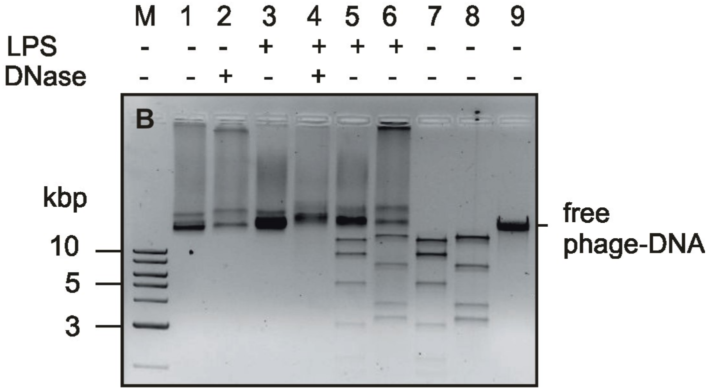

Figure S1: Ethidium bromide stained 1% agarose gel electrophoresis with 5×10^9 pfu of HK620 (lane 1), treated with 0.1 mg ml^{-1} desoxyribonuclease (lanes 2 and 4) or with 0.2 mg ml^{-1} LPS from *E. coli* H TD2158 (lanes 3 to 6). Released phage DNA was tested for accessibility to HindIII (lane 5) or EcoRI (lanes 6). As controls purified HK620 DNA (lane 9) was digested by HindIII and EcoRI (lanes 7 and 8).

1.6×10^{11} Pfu ml^{-1} of phage HK620 were incubated overnight with 0.2 mg/ml LPS from *E. coli* H TD2158 at 37 °C in standard buffer. Afterwards the ejection products were analyzed on agarose gels as described before [21]. A mixture of HK620 virions with purified LPS from its host *E. coli* H TD2158 showed a significantly increased amount of free DNA (lane 3), that could be digested with DNase (lane 4). Restriction fragments were the same as observed for digests of free DNA and also fully resolvable as low molecular weight bands (data not shown). After LPS treatment, a portion of the DNA remained protected from DNase and restriction enzymes (lanes 4-6). No broad DNA band distributions occurred that would result from partially ejected particles, but only an increase of the distinct full length DNA band was observed. Thus, only a part of the particles in the preparation released their DNA, but those that were triggered showed full release. Free DNA of HK620 migrated more slowly in agarose gels agreement with the large genome size of 38.3 kbp (lane 9), showed a characteristic fragmentation pattern with restriction enzymes EcoRI and HindIII (lanes 7 and 8) and was fully accessible to DNase (data not shown). By contrast, DNA encapsulated inside phage HK620 virions appeared as multiple slowly migrating bands not accessible to DNase (lane 2). Contamination by free, spontaneously released DNA in the phage preparations (lane 1) disappeared after DNAse treatment (lane 2).

Figure S3: LPS-mediated DNA-release from phage HK620 observed *in vitro* at temperatures above 45 °C.

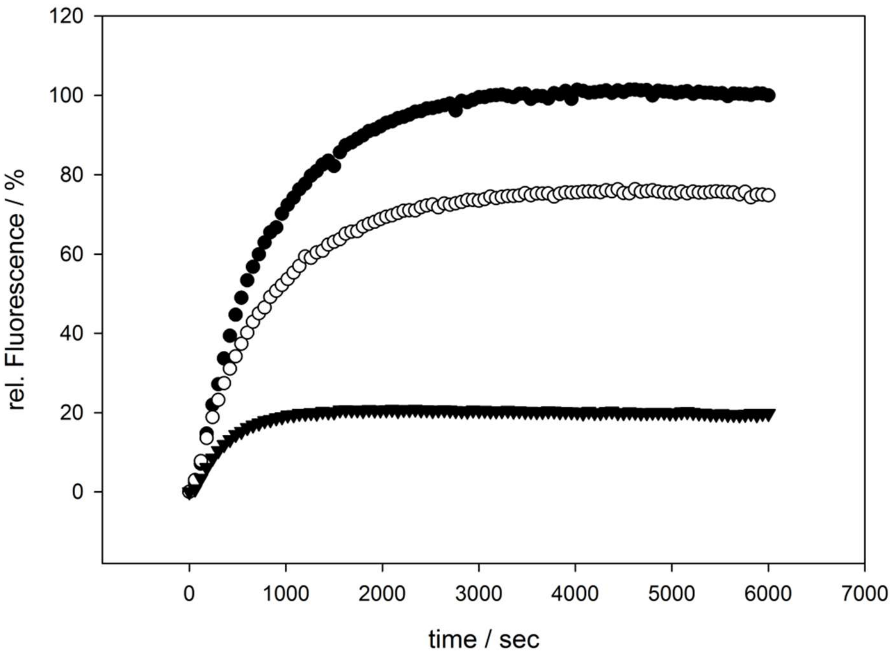

Figure S2: DNA ejection from phage HK620 (6.7 · 109 pfu) triggered by 16.7 µg/ml E. coli H TD2158 LPS in the presence of the fluorescent DNA-binding dye Yo-Pro at 45 °C (black circles), 48 °C (white circles) and 50 °C (black triangles).

Figure S4: Temperature dependencies of HK620 DNA ejection rate constants

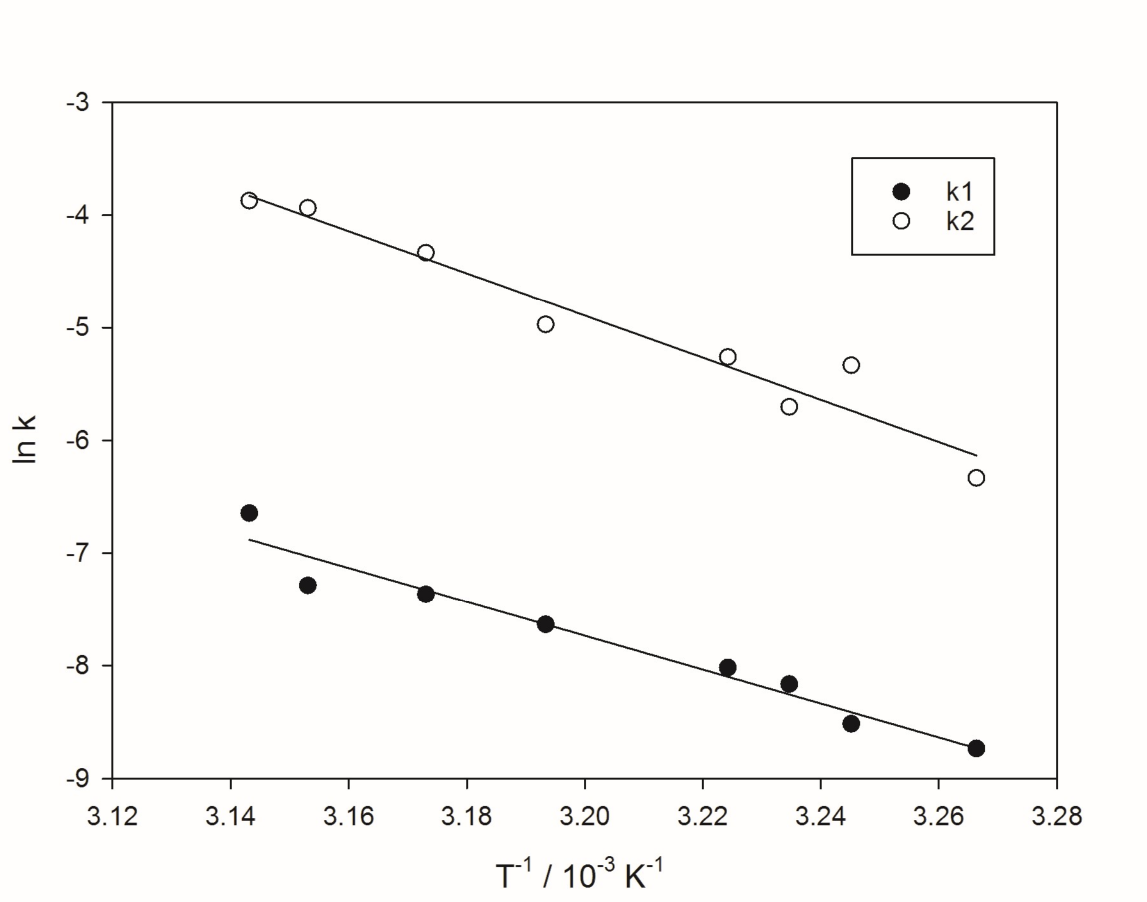

Figure S2. Arrhenius plot of temperature dependencies of HK620 DNA ejection rate constants. Velocities were analyzed with fluorescence spectroscopy and the resulting two first-order rate constants determined at different temperatures (*cf.* Figure 1). Slopes determined from linear regression were 125 kJ mol^{-1} and 156 kJ mol^{-1}.

Figure S5: O18 serogroups

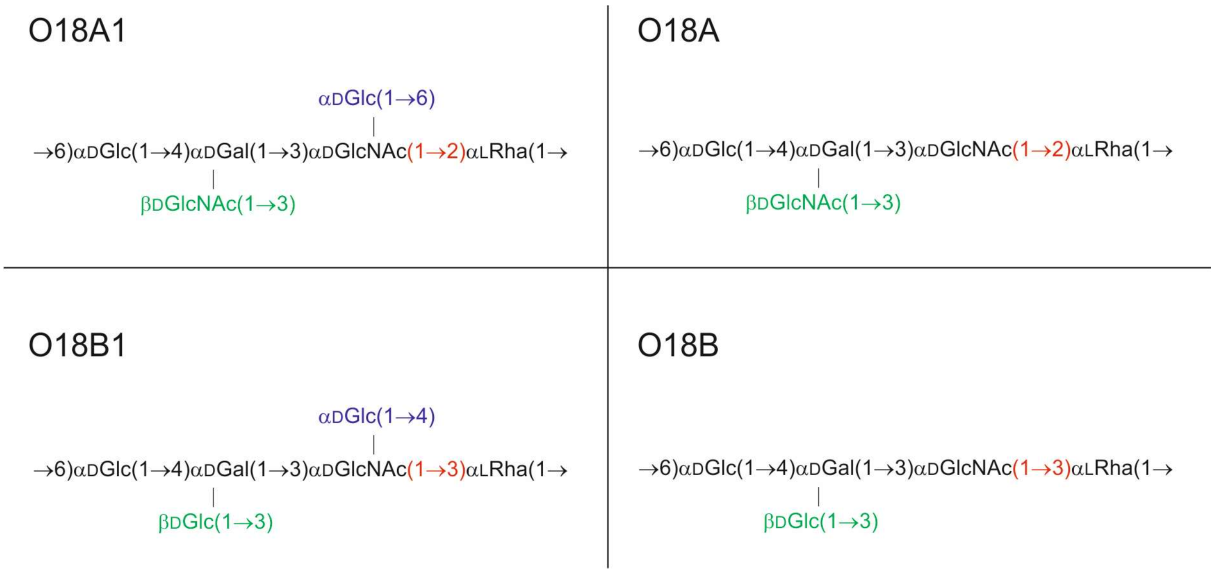

Figure S3: Composition of *E. coli* O-serogroups of type O18 according to [38].

Figure S6: Comparison of DNA ejection velocities of O-antigen specific podoviruses HK620, P22 and Sf6

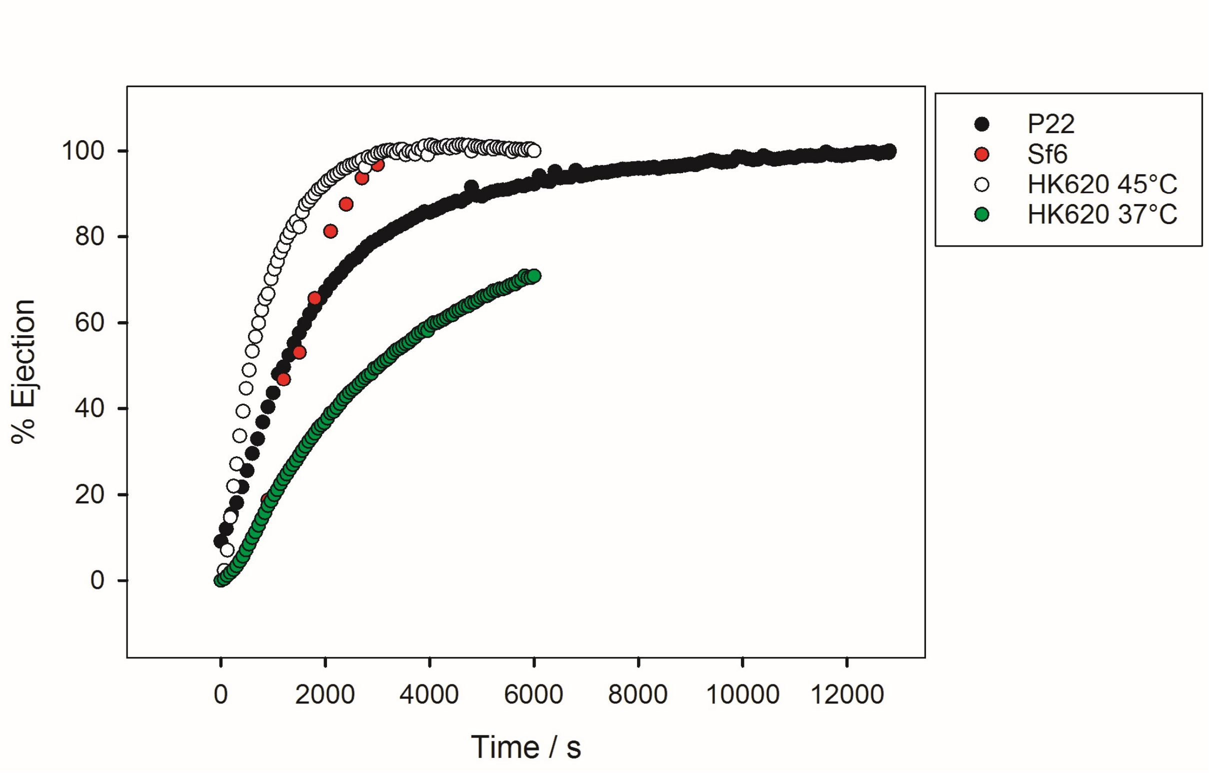

Figure S4: DNA ejection from podoviruses P22 [21] or HK620 (this work) was quantified via DNA fluorescence. For Sf6, kinetics were adapted from [35].

Figure S7: Dimensions of glycolipid receptors and tail protein components

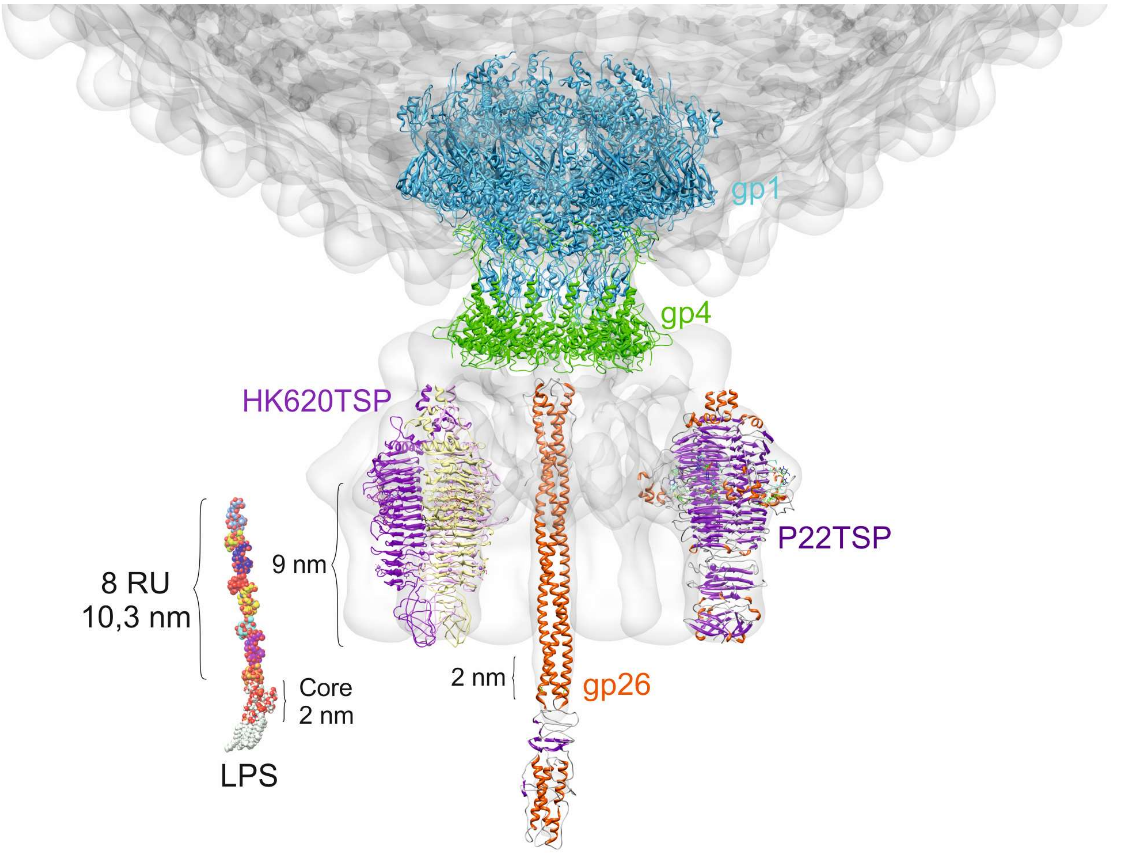

Andres et al. designed this model for phage P22 by fitting known structural elements of the phage into experimental electron density of the P22 cryo-EM structure (EMDB: 1222) [19,21]. Due to missing structural data the P22 model was used as a basis for the phage HK620 particle in the figure. HK620TSP was set on the height of P22TSP (purple, PDB: 1TSP) [20]. The plug protein gp26 is shown in orange (PDB: 2POH) as well as the portal struc ture of gp1 and gp4 (PDB: 3LJ4). For constructing the LPS structural model, known components were linked [16,38]. A stretched O-polysaccharide chain was assumed.
